# Comparative vector competence of post-2015 St. Louis encephalitis virus in *Culex tarsalis* and *Culex quinquefasciatus* mosquitoes

**DOI:** 10.64898/2025.12.16.694562

**Published:** 2025-12-16

**Authors:** M. Arturo Flores Rodriguez, Hongwei Liu, Erik Turner, Rochelle Leung, Sunny An, Lark L. Coffey

**Affiliations:** 1 Department of Pathology, Microbiology, and Immunology, School of Veterinary Medicine, University of California, Davis, Davis, CA, USA

## Abstract

The human pathogenic orthoflavivirus St. Louis encephalitis virus (SLEV) reemerged in the western United States in 2015 after more than a decade of apparent absence and has since expanded throughout California with sustained, interannual transmission. This shift from the historically sporadic pattern of SLEV activity prior to 2003 raises the question of whether contemporary SLEV strains differ in fitness in *Culex* vectors compared with earlier strains. To determine whether reemerging SLEV possess augmented infectivity and transmissibility that may have facilitated reestablishment, we compared the vector competence of five genotype III SLEV strains detected in California between 2016 and 2023 with a historic genotype V strain from 2003. Laboratory colonies of the two primary California vectors, *Culex (Cx.) tarsalis* and *Cx. quinquefasciatus*, were orally exposed to bloodmeals containing 3, 5, or 6.7 log_10_ plaque forming units (PFU)/mL of SLEV, and infection, dissemination, and transmission were assessed 13,14, or 15 days later by quantifying SLEV RNA in individual mosquitoes. Both species exhibited strong dose-dependent responses, with minimal infection at 3 log_10_ PFU/mL and uniformly high infection, dissemination, and saliva positivity at 6.7 log_10_ PFU/mL. At 5 log_10_ PFU/mL, genotype III strains infected *Cx. quinquefasciatus* more efficiently than the historical 2003 strain, which failed to infect this species. In *Cx. tarsalis*, fitness differences among SLEV strains were more modest and strain-specific. These findings demonstrate that multiple genotype III SLEV strains exhibit equal or greater vector infectivity in *Cx. quinquefasciatus* than the 2003 genotype V strain, suggesting that enhanced fitness in this vector may contribute to the persistence and geographic spread of SLEV in California since its reemergence and underscoring the need for continued vector surveillance and targeted control efforts to reduce SLEV transmission to humans.

## AUTHOR SUMMARY

St. Louis encephalitis virus (SLEV) is a mosquito-borne virus that can cause neurologic disease in humans. After being absent for more than a decade, SLEV reappeared in California in 2015 and has since spread across the state. We wanted to understand whether the newly circulating SLEV strains are better able to infect and be transmitted by local mosquitoes than older strains, which could help explain why the virus has reestablished. In the laboratory, we exposed two common California vector species, *Culex (Cx.) tarsalis* and *Cx. quinquefasciatus*, to recent SLEV strains from 2016–2023 and to a historic 2003 strain. We found that the recent strains infected *Cx. quinquefasciatus* mosquitoes more efficiently than the historic strain, while differences in *Cx. tarsalis* were smaller and varied by strain. These results suggest that enhanced mosquito infectivity of contemporary SLEV strains may have contributed to the persistence and spread of SLEV in California. Our findings highlight the importance of ongoing mosquito surveillance and targeted control to reduce the risk of SLEV transmission to humans.

## INTRODUCTION

St. Louis encephalitis virus (SLEV, *Flaviviridae, Orthoflavivirus louisense*) circulates in an enzootic cycle between birds and mosquitoes in the Americas and can spill over into humans, causing febrile illness and sometimes encephalitis. SLEV produced periodic outbreaks in the United States beginning in the 1930s and was a major cause of encephalitis during the 1970s (1, 2), including in California from the 1940s through the 1990s (3, 4). Because no specific treatment or vaccine for SLEV exists, mosquito control remains the primary public health tool for limiting SLEV transmission. In California, vector control agencies monitor arbovirus activity using multiple approaches, including mosquito surveillance. Such surveillance showed near annual SLEV activity from 1970 until 2003 (3), the year the related flavivirus West Nile virus (WNV) invaded the state (5). Starting the next year (2004) until 2015, SLEV was not detected in California mosquitoes. In 2015, a SLEV outbreak occurred in Phoenix, Arizona, concurrent with a WNV epidemic (6). That same year, SLEV was detected again in California and subsequently spread northward from southern California into the Central Valley and farther north from 2016–2025 (7) into 22 of 58 (38%) of California counties. Since 2015, SLEV-positive mosquito pools have included *Culex (Cx.) stigmatosma, pipiens, tarsalis*, and *quinquefasciatus* (8), the latter three of which were previously identified as the primary vectors of both WNV and historic SLEV in California (9–12). Between 2015–2025, 95% of SLEV positive pools in California were *Cx. tarsalis* or *Cx. quinquefasciatus* (8). The geographic expansion, increasing number of human cases most years, and detection of SLEV positive mosquito pools since 2015, shows SLEV is becoming increasingly prevalent in California (8). This recent pattern contrasts with historical pre-2003 SLEV activity, during which the virus was typically locally introduced, amplified for a 1–2 year period, and then disappeared (4, 13–15). Moreover, although SLEV and WNV now co-circulate in many of the same California counties, SLEV remains largely absent from WNV hotspots in the north and south and is instead more common in the Central and Coachella Valleys (7). The interannual persistence and geographic spread of reintroduced SLEV since 2015 raises the question of whether its reestablishment is mediated by increased fitness, defined here as infectivity and transmissibility, in the two primary California mosquito vector species *Cx. tarsalis* and *Cx. quinquefasciatus*.

Our analyses comparing SLEV genomes in the western United States from 2015–2018 with publicly available sequences show that these strains are most closely related to SLEV associated with an outbreak in Argentina in 2005 (16) and to a 2014 isolate from Argentine mosquitoes (17), supporting a South American introduction (18, 19). SLEV circulating in California and the western United States from 2014–2018 forms at least 4 geographically distinct clusters (a-d), all belonging to genotype III (19). Subsequent SLEV phylogenetic studies conducted after 2018 indicate that multiple SLEV importations have occurred in California, Arizona, and other western states, yet all introduced genomes belong to genotype III (20). Despite these genetic insights, no phenotypic studies in mosquito vectors have characterized infection or transmission dynamics of reemerging SLEV, nor compared post-2015 strains with pre-2004 SLEV that historically circulated in California and which has been extensively evaluated in mosquito and avian hosts (14, 21–27). For context, WNV, which shares many avian reservoirs and *Cx.* vector species with SLEV, has evolved to increase transmission competence in *Cx.* spp. across the US since 2002 (28). Work in California further showed that WNV strains from later outbreaks (2004–2012) outcompete a 2003 strain in house finches but not in *Cx. tarsalis* (29, 30), suggesting that enhanced avian rather than mosquito fitness likely contributed to WNV amplification to outbreak levels during a decade of circulation in California. Although WNV evolution in both vectors and avian hosts across the US and in California is well documented, comparable studies for SLEV are lacking, leaving unanswered whether changes in viral fitness contributed to SLEV reemergence in California after 2015. To address this gap, the goal of this study was to evaluate the extent to which SLEV reemergence may be driven by altered infectivity and transmissibility in two primary California mosquito vectors. We therefore evaluated infection, dissemination, and transmission success in *Cx. tarsalis* and *Cx. quinquefasciatus* that ingested bloodmeals containing one of three doses of SLEV from either 2003 or 2016–2023.

## MATERIALS AND METHODS

### Biosafety.

All work with SLEV was performed in a biosafety level 3 laboratory at the University of California, Davis, under an approved Biological Use Authorization (#R1863).

### Cell culture.

African green monkey kidney Vero (ATCC CCL-81) cells used for SLEV isolation from mosquito pools and bloodmeal titrations were maintained in Dulbecco’s Modified Eagle’s Medium (DMEM) supplemented with 5% fetal bovine serum (FBS), 100 U/mL penicillin and 100 μg/mL streptomycin at 37°C with 5% CO_2_ in a humidity-controlled incubator.

### SLEV activity in California mosquitoes.

The total annual number of SLEV positive mosquito pools and SLEV detections in each mosquito species in California were sourced from the California Department of Public Health Vector Borne Disease Section Annual Reports from 2015–2024 (8). For the 2016 report, detailed data on the numbers of SLEV detections in different mosquito species were not provided; this data was instead derived from weekly bulletins from 2016 (31). The 2025 data was obtained from the WestNile.ca.gov website (32) and is current as of December 8, 2025.

### SLEV isolations from mosquito pools.

This study focuses on the period 2016–2023 and utilizes five genotype III SLEV strains isolated from California locations with ongoing mosquito pool activity compared to a historical genotype V strain from 2003. Pools of *Cx. tarsalis* and *Cx. quinquefasciatus* collected in California (Table 1) and confirmed SLEV positive by an established qRT-PCR method (33) were obtained through routine arbovirus surveillance conducted by mosquito abatement districts in California and the Davis Arbovirus Research and Training laboratory at the University of California, Davis. SLEV positive mosquito pools were centrifuged at 4,000 g for 5 minutes to pellet debris and then filtered through a 0.22 μM filter (Millex GP, Millipore, Billerica, MA, USA). The filtrate was inoculated into confluent T25 Vero cell monolayers and incubated for 4 days. Virus recovered from culture supernatants was subsequently passaged 2–4 additional times on Vero cells to generate working stocks, which were titrated and stored at −70°C in aliquots until use. The genotype V SLEV strain isolated in 2003, representing the lineage historically circulating in California prior to 2004, was included as a comparator; this strain had been isolated previously and was re-sequenced since 2 additional Vero cell passages were performed to generate a working stock. The genotype III strains share ≈99% nucleotide and amino identity genome-wide with one other and ≈93% nucleotide and ≈98% amino acid identity with the 2003 genotype V strain (Table 2).

### SLEV titrations.

Titers of SLEV working stocks and bloodmeals were quantified by plaque assays using Vero cells. Serial tenfold dilutions of virus were absorbed onto confluent monolayers for 1 hour at 37°C with 5% CO_2_. Following adsorption, an agarose-based overlay containing nutrient medium, 0.5% agarose, 3% bicarbonate (Sigma Aldrich, St. Louis, MO, USA) was added, and after 5 days of incubation at 37°C and 5% CO_2_ a second overlay containing additional 3% neutral red (Sigma Aldrich, St. Louis, MO, USA) was applied. On day 7 visible plaques were enumerated and the titer was determined and reported in log_10_ plaque forming units (PFU)/mL. Each stock or bloodmeal titration was performed in duplicate, and the mean value is reported.

### Mosquitoes.

Laboratory colonies of *Cx. tarsalis* and *Cx. quinquefasciatus* were used. The *Cx. tarsalis* colony originated from collections in 2004 at the Kern National Wildlife Refuge in California. The *Cx. quinquefasciatus* colony originated from collections in Los Angeles, California in 2016. Both colonies have been maintained continuously since establishment. Mosquitoes were reared under insectary conditions of 22°C, ~30% relative humidity, and 12 hour:12 hour light:dark cycle. Larvae were maintained in 1 liter deionized water at 200–400 larvae per pan and fed one pinch of fish food (Tetra, Melle, Germany) every other day until pupation. Adults were housed in 30 × 30 × 30 cm cages (BugDorm, Megaview Science, Taiwan) with constant access to 10% sucrose. Adults aged 3–7 days were used for vector competence experiments.

### Vector competence.

A total of 5 genotype III SLEV strains were assessed; three were tested in *Cx. tarsalis* and all were tested in *Cx. quinquefasciatus*. The historical 2003 genotype V strain was included for comparison in both species. Approximately 200 mixed-sex mosquitoes were aspirated from colony cages one day prior to bloodfeeding and transferred into quart-sized plastic containers with mesh lids and access to 10% sucrose. SLEV stocks were diluted in heparinized sheep blood (HemoStat Laboratories, Dixon, CA, USA) to target doses of 3, 5, and 6.7 log_10_ PFU/ml. Bloodmeals were offered for 60 minutes using a collagen membrane mounted on a Hemotek member feeder (Hemotek Ltd, Blackburn, United Kingdom) maintained at 37°C. Fully engorged females, identified by visible blood in the abdomen, were anesthetized for 20 seconds with CO_2_, sorted into clean plastic containers at a density of 30–60 mosquitoes, and held at 28°C with 60–70% humidity and 12 hour:12 hour light:dark cycle for 13, 14, or 15 days, with constant access to 10% sucrose. A total of 6 experiments were conducted in which 2–4 SLEV strains were compared at matched bloodmeal titers. Each strain was tested in 1–4 experiments; the combined data from all experiments is shown.

At 13, 14, or 15 days post-bloodfeeding, mosquitoes were CO_2_-anesthetized and placed immobile on a chill table for dissection. Legs and wings were removed prior to saliva collection, which was performed by inserting the proboscis into capillary tubes containing FBS for 20–30 minutes. Each capillary tube was placed in a 1.5 mL tube containing 100 μL DMEM and centrifuged at 8,000 g for 1 minute to recover saliva. Legs & wings and bodies were placed into 2 mL tubes (Thermo Fisher Scientific, Emeryville, CA) containing 250 μL DMEM and a 5 mm glass bead (Thermo Fisher Scientific, Emeryville, CA). Dissection tools were rinsed once in 70% ethanol between each sample to prevent cross-contamination. Tissues were homogenized at 30 Hz for 4 minutes in a TissueLyser (Retsch, Haan, Germany) and centrifuged at 10,000 g for 2 minutes, then stored at −70°C until processing.

### SLEV RNA detection, enumeration, and reporting.

Tissues were thawed and viral RNA was extracted from a portion of bodies and legs & wings using the MagMax Viral RNA Extraction Kit (ThermoFisher Scientific, Emeryville, CA, USA) or saliva using Trizol LS (ThermoFisher). For Trizol extractions, 400 μL TRIzol LS and 100 μL chloroform was added to 100 μL of saliva. The tube was mixed vigorously and then centrifuged for 5 minutes at 20,000 g at 4°C. The clear upper aqueous layer was transferred to a new 1.5 mL tube, and 200 μL of isopropanol was added. Then the tube was gently mixed by inverting 5–10 times followed by incubation for 30 minutes at −20°C. The sample was centrifuged for 15 minutes at 20,000 g at room temperature. The supernatant was discarded, and the sample was centrifuged for 30 seconds, and residual ethanol was removed using a 20 μL pipette. Total RNA isolated was suspended in 90 μL buffer following the manufacturer’s instructions (tissue samples) or 100 μL of nuclease-free water (saliva) from the Trizol method. SLEV RNA in mosquito bodies, legs & wings, and saliva was quantified by qRT-PCR using a TaqMan Fast Virus 1-Step Mastermix and a SLEV-specific primer set at 20 μM (Table 3) using the following reagents: 3.2 μl TE buffer, 1.8 μl of the primer and probe mix, 5 μl of Fast mix, and 10 μl of RNA. The cycling conditions were as follows: 50°C for 5 minutes, 95°C for 20 seconds, and 40 cycles of 95°C for 3 seconds followed by 60°C for 30 seconds. Cycle threshold (Ct) values were converted to RNA genome copies using standard curves generated from known SLEV RNA concentrations included on each qRT-PCR plate. Negative control samples lacking SLEV RNA were also included in each run. Samples were assayed in technical duplicates or triplicates and averaged after conversion to RNA copies per mL. Samples in which only one replicate produced a Ct < 40 were considered negative. The limit of detection (LOD) was defined as the mean Ct for the least concentrated standard curve point producing a Ct < 40 across all runs. Samples that did not yield a detectable Ct < 40 are not included in group means for titer measurements. SLEV RNA concentrations are reported as log_10_ genomes/tissue or saliva sample. Infection (bodies), dissemination (legs & wings), and transmission (saliva) rates are presented as the proportion of positive samples out of the total number tested for each tissue type, with denominators for legs & wings and saliva restricted to mosquitoes with positive bodies or legs & wings, respectively.

### SLEV genetic comparisons.

Alignments of SLEV genomes were generated to compare nucleotide and amino acid identity using the EMBL-EBI MAFFT multiple sequence alignment tool and CLUSTAL (34).

### Statistical Analyses.

All statistical analyses were performed in GraphPad Prism version 10.3.1. Analyses of infectious SLEV titers (PFU) and SLEV RNA levels were conducted on log-transformed values. Fisher’s exact tests with Benjamini–Hochberg correction were used to compare rates of infection (bodies), dissemination (legs & wings), and transmission (saliva). For RNA quantification, differences in mean RNA levels among strains were evaluated using ANOVA with Šídák’s multiple-comparisons test. Only adjusted p values ≤ 0.05 were considered statistically significant. The specific statistical tests used for each comparison are provided in the Results section.

### Data availability.

All data supporting the findings of this study are available from the corresponding author upon request.

## RESULTS

### Dose response.

**Figure 1:**
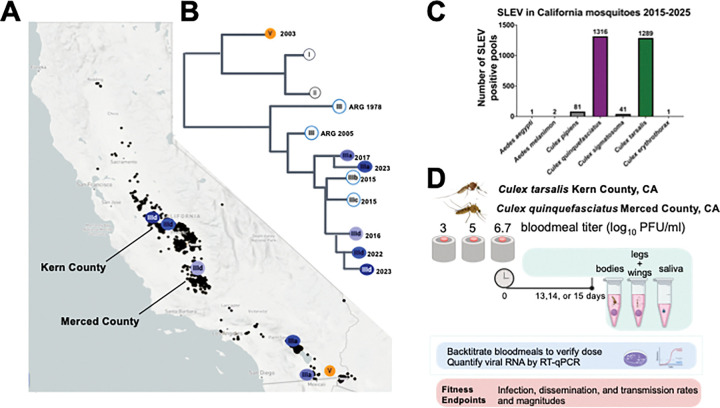
(**A**) Map of California showing locations of SLEV detections in mosquito pools from 2015–2025. Data from (7). The sources of the SLEV strains used in this study are shown in the colored circles and correspond to the labels on the phylogenetic tree. ARG indicates Argentina. (**B**) SLEV positive mosquito pools in California from 2015–2025 by species. 2025 data is current as of December 8, 2025. Data from (8). (**C**) Experimental design for SLEV vector competence studies using *Culex tarsalis* and *Culex quinquefasciatus* from California. Mosquitoes that ingested bloodmeals containing 3, 5, or 6.7 log_10_ PFU of different SLEV strains were incubated for 13, 14, or 15 days and then dissected to assess infection (bodies), dissemination (legs & wings), and transmission (saliva) by qRT-PCR. Bloodmeals were back-titrated using Vero cell plaque assays to verify the administered dose.

**Figure 2:**
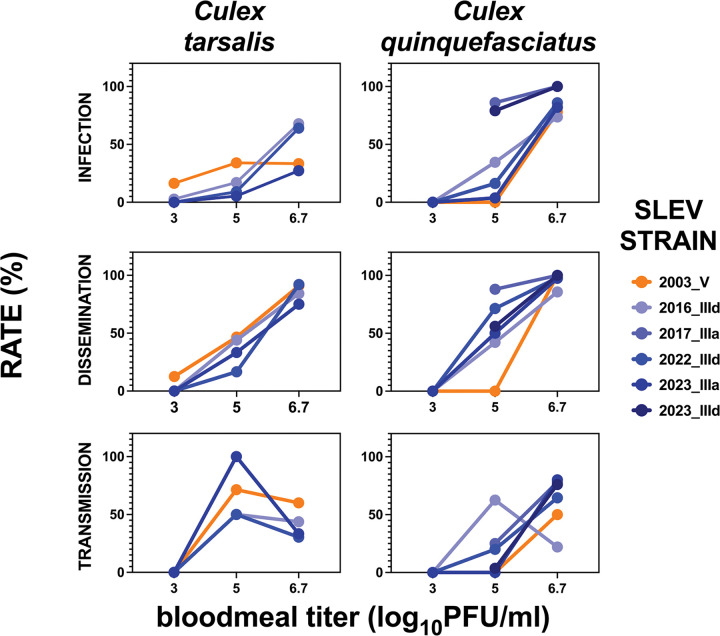
Dose response to SLEV represented as infection (bodies), dissemination (legs & wings), and transmission (saliva) rates 13, 14, or 15 days post-bloodmeal in cohorts of *Culex tarsalis* and *Culex quinquefasciatus* mosquitoes that ingested bloodmeals containing different SLEV strains. PFU is plaque forming units.

We assessed the vector competence of five genotype III SLEV strains isolated from different areas of California (Figure 1A) and compared them with each other and a historical 2003 SLEV strain representing genotype V (Figure 1B) that circulated in California prior to 2004. All strains were isolated from SLEV positive mosquito pools and minimally passaged in Vero cells (Table 1). We selected *Cx. tarsalis* and *Cx. quinquefasciatus* for evaluation because these species most frequently tested positive for SLEV in California, representing 47% (1289/2731) and 48% (1316/2731) respectively, of SLEV mosquito pool detections statewide from 2015–2025 (Figure 1C). Mosquitoes from both species were offered bloodmeals containing one of three SLEV doses, 3, 5, or 6.7 log_10_ PFU/ml (Figure 1D). Infection (bodies), dissemination (legs & wings), and transmission (saliva) outcomes displayed strong dose response to bloodmeal titer (Figure 2). At 3 log_10_ PFU/mL, infection rates were either 0% or low in both species, and dissemination or transmission did not exceed 16%. In contrast, infection, dissemination, and transmission increased when mosquitoes ingested blood containing 5 or 6.7 log_10_ PFU/mL SLEV. Back-titration confirmed that delivered bloodmeal doses were within ±0.5 log_10_ PFU/mL of the intended concentrations.

### Vector species comparisons.

**Figure 3:**
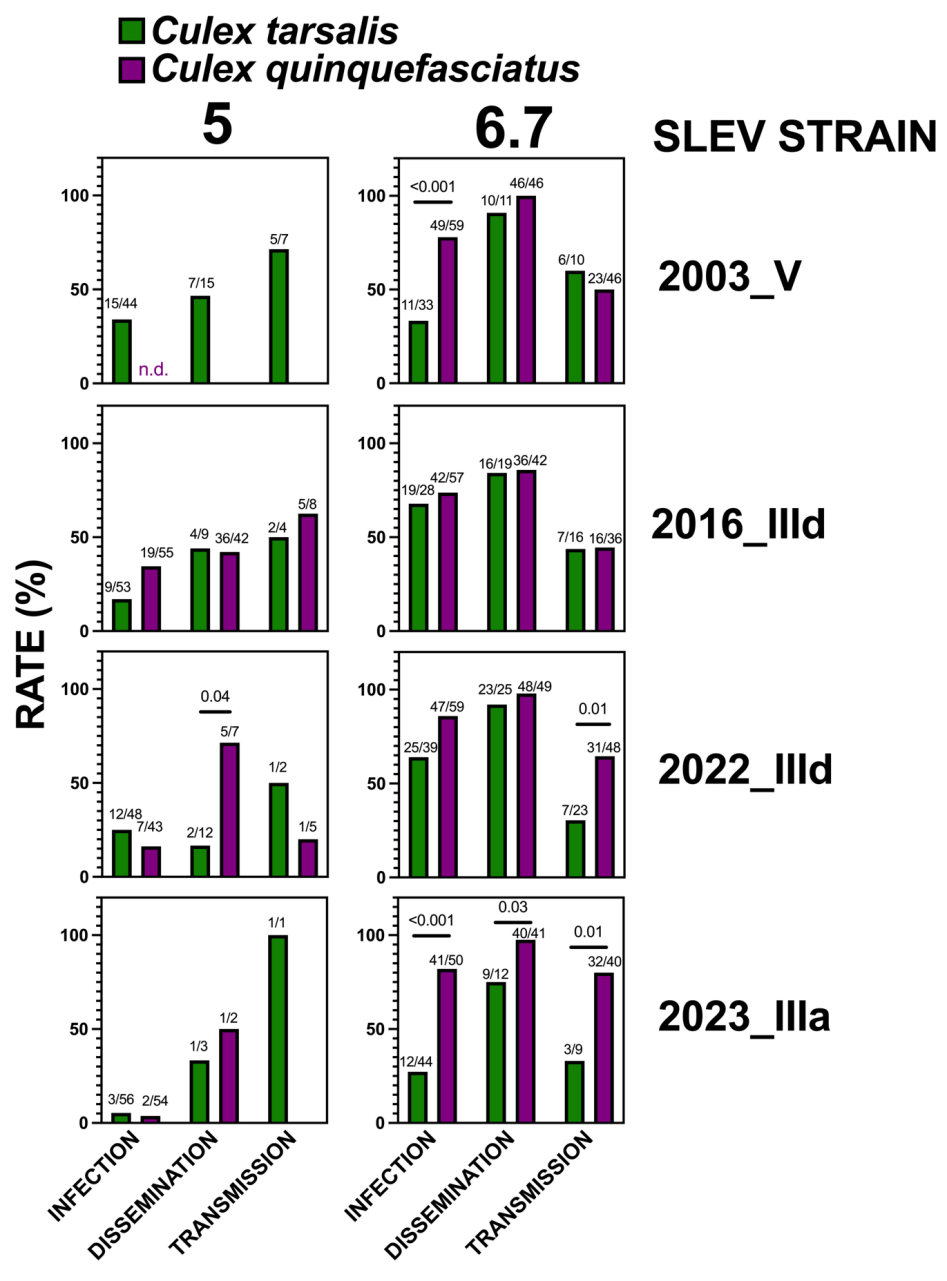
SLEV infection (bodies), dissemination (legs & wings), and transmission (saliva) rates by species 13,14, or 15 days post-bloodmeal in cohorts of *Culex tarsalis* and *Culex quinquefasciatus* mosquitoes that ingested 5 or 6.7 log10 PFU/mL of different SLEV strains. Rates were not statistically significantly different unless indicated with a p-value (Fisher’s exact tests; only values p ≤ 0.05 were considered significant). Ratios above bars show positives out of total tested for each sample type. n.d. indicates none detected.

We compared the relative fitness of SLEV in the two mosquito species (Figure 3). At a bloodmeal dose of 5 log_10_ PFU/ml, *Cx. quinquefasciatus* showed no susceptibility to the 2003 genotype V strain, whereas *Cx. tarsalis* exhibited infection, dissemination, and transmission rates of 34% (15/44), 47% (7/15), and 71% (5/7), respectively. In contrast, for the genotype III strains, infection, dissemination, and transmission rates at 5 log_10_ PFU/mL were generally similar between the two species, with the exception of 2022_IIId, which displayed significantly higher dissemination in *Cx. quinquefasciatus* [71% (5/7) versus 15% (2/12), p = 0.04, Fisher’s exact test]. At 6.7 log_10_ PFU/mL, infection rates of the 2003 genotype V strain were significantly higher in *Cx. quinquefasciatus* compared to *Cx. tarsalis* [85% (49/59) versus 33% (11/33), p < 0.001, Fisher’s exact test], and transmission of 2022_IIId was also significantly greater in *Cx. quinquefasciatus* [65% (31/48) versus 30% (7/23), p < 0.01]. For the 2023_IIIa strain at 6.7 log_10_ PFU/ml, rates of infection [82% (41/50) versus 27% (12/44), p < 0.001], dissemination [97% (40/41) versus 75% (9/12), p = 0.03], and transmission [80% (32/40) versus 33% (3/9), p = 0.01, all Fisher’s exact test] were all significantly higher in *Cx. quinquefasciatus*. Overall, these results indicate that the genotype III SLEV strains tested exhibit comparable or greater fitness in *Cx. quinquefasciatus* than in *Cx. tarsalis*. At 5 log_10_ PFU/mL, genotype III viruses show higher fitness in *Cx. quinquefasciatus* than the historical genotype V comparator strain.

### SLEV strain comparisons in *Cx. tarsalis*.

**Figure 4:**
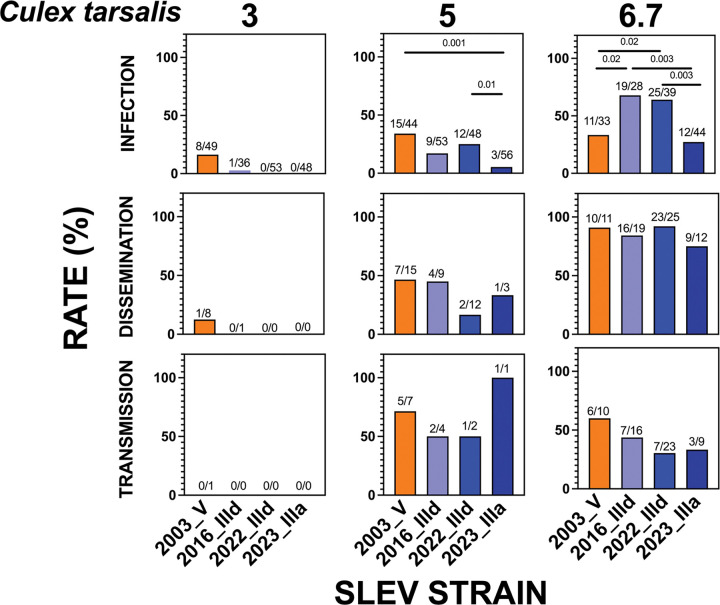
SLEV infection (bodies), dissemination (legs & wings) and transmission (saliva) rates 13,14, or 15 days post-bloodmeal in cohorts of *Culex tarsalis* mosquitoes that ingested 3, 5, or 6.7 log_10_ PFU/mL of different SLEV strains. Rates were not statistically significantly different unless indicated with a p-value (Fisher’s exact tests; only values p ≤ 0.05 were considered significant). Ratios above bars show positives out of total tested for each sample type.

We next compared the relative fitness of SLEV strains in *Cx. tarsalis* (Figure 4, Table 4). At 3 log_10_ PFU/mL, all strains produced either no or low infection rates, and dissemination and transmission were similarly rare. At 5 log_10_ PFU/mL, infection rates of the 2003 genotype V strain were significantly higher than those of 2023_IIIa [34% (15/44) versus 5% (3/56), p = 0.001, Fisher’s exact test], although infection rates for 2003_V did not differ from those of 2016_IIId or 2022_IIId. Dissemination and transmission rates at this dose did not differ significantly among strains. However, infection rates of 2022_IIId were significantly higher than 2023_IIIa [25% (12/48) versus 5% (3/56), p = 0.01, Fisher’s exact tests].

At 6.7 log_10_ PFU/mL, infection rates of 2016_IIId [68% (19/28)] and 2022_IIId [64% (25/39)] were significantly higher than 2003_V [33% (11/33), both p = 0.02, Fisher’s exact test] and 2023_IIIa [27% (12/44), p = 0.003 for each, Fisher’s exact test]. No significant differences were observed in dissemination or transmission rates at this dose. Across doses, the two IIId lineage strains (2016_IIId and 2022_IIId), exhibited similar fitness profiles in all tissues. Overall, these findings show strain-specific differences in SLEV fitness in *Cx. tarsalis*, with some but not all genotype III strains demonstrating higher infection rates than the historical genotype V strain, but without consistent differences in dissemination or transmission.

### SLEV strain comparisons in *Cx. quinquefasciatus.*

**Figure 5:**
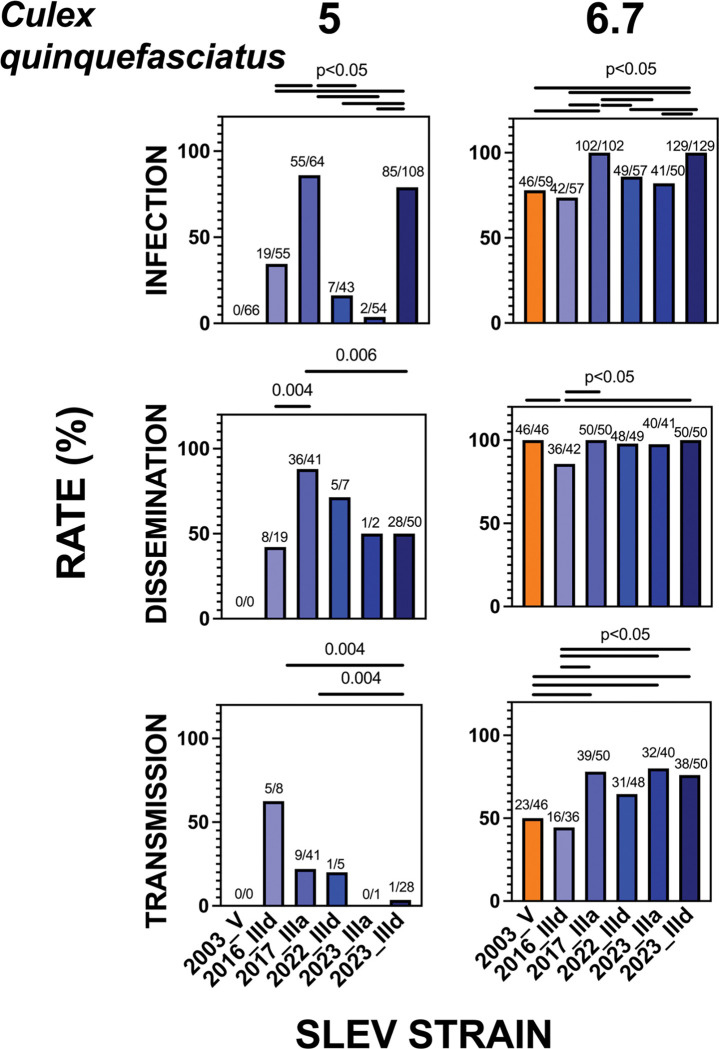
SLEV infection (bodies), dissemination (legs & wings) and transmission (saliva) rates 13,14, or 15 days post-bloodmeal in cohorts of *Culex quinquefasciatus* mosquitoes that ingested 3, 5, or 6.7 log_10_ PFU/mL of different SLEV strains. Rates were not statistically significantly different unless indicated with a p-value (Fisher’s exact tests; only values p ≤ 0.05 were considered significant). Ratios above bars show positives out of total tested for each sample type. For 2017_IIIa and 2023_IIId cohorts, only legs & wings and saliva from a subset of positive bodies were tested.

We next assessed relative fitness of SLEV strains in *Cx. quinquefasciatus* (Figure 5, Table 5). At 5 log_10_ PFU/ml, no mosquitoes became infected after ingesting the 2003 genotype V strain. In contrast, all five genotype III strains produced infection, ranging from 4% to 85%. At this dose, infection rates of 2017_IIIa [85% (55/64)] and 2023_IIId [78% (85/108)] were significantly higher than those of 2016_IIId [34% (19/55)], 2022_IIId [16% (7/43)], and 2023_IIIa [4% (2/54), p < 0.05, Fisher’s exact tests]. Although dissemination rates for 2016_IIId [88% (36/41)] were higher than those for 2016_IIId [42% (8/19)] and 2023_IIId [56% (28/50)m=, p < 0.05], transmission rates were significantly greater for 2016_IIId [63% (5/8)] and 2017_IIIa [22% (9/41)] compared to 2022_IIId [20% (1/5) or 2022_IIId [3% (1/28), p < 0.05, Fisher’s exact tests].

At 6.7 log_10_ PFU/mL, most mosquitoes exposed to any SLEV strain became infected, and numerous statistically significant differences were observed. The 2017_IIIa and 2023_IIId strains, which infected 100% of tested bodies (102/102 and 129/129, respectively), produced significantly higher infection rates than 2003_V [78% (46/59)], 2016_IIId [74%, (42/57)], 2022_IIId [86% (49/57)], and 2023_IIIa [82% (41/50, all p < 0.05, Fisher’s exact tests). At this dose, a high proportion of infected bodies also developed disseminated infections in legs and wings (86–100%), and saliva infection was also frequently detected (34–78%). Dissemination rates were significantly higher for 2003_V [100% (46/46)], 2017_IIIa [100% (50/50)], and 2023_IIId [100% (50/50] compared to 2016_IIId [86% (36/42)], p < 0.05, Fisher’s exact tests). Transmission rates for 2017_IIIa [78% (39/50)], 2023_IIIa [80% (32/40)], and 2023_IIId [95% (38/50)] were significantly higher than those for 2003_V [50% (23/46)] and 2016_IIId [44% (16/36), p < 0.05, Fisher’s exact tests].

In contrast to *Cx. tarsalis*, where strains within the same subgenotype (IIIa or IIId) showed similar fitness, *Cx. quinquefasciatus* exhibited variable and dose-dependent infection patterns. For the IIId strains, the order of infection rates differed by dose (2023_IIId > 2016_IIId > 2022_IIId at 5 log_10_ PFU/mL versus 2023_IIId > 2022_IIId > 2016_IIId at 6.7 log_10_ PFU/mL). Between the IIIa strains, infection rates at 5 log_10_ PFU/mL differed markedly, with 2023_IIIa infecting 4% (2/54) and 2017_IIIa infecting 86% (55/64) of tested bodies (p < 0.05, Fisher’s exact tests). There was also no clear trend of increasing infection rates associated with the year a strain was detected.

Overall, genotype III SLEV strains demonstrated high and strain-specific fitness in *Cx. quinquefasciatus*, with several strains infecting and transmitting more efficiently than the historical 2003 genotype V strain.

### SLEV genetic determinants of vector fitness.

We compared genome-wide amino acid differences across strains (Table 6) to evaluate whether variant amino acids are shared between strains with similar vector fitness (e.g. 2017_IIIa and 2023_IIId that efficiently infect *Cx. quinquefasciatus*). Of 60 amino acid differences across any of the 6 strains, 38 were unique to the 2003 V strain. There were no shared amino acid differences in strains with high (2017_IIIa and 2023_IIId) or low (2016_IIId, 2022_IIId, 2023_IIIa) *Cx. quinquefasciatus* infection patterns.

### SLEV RNA levels in mosquito tissues.

**Figure 6:**
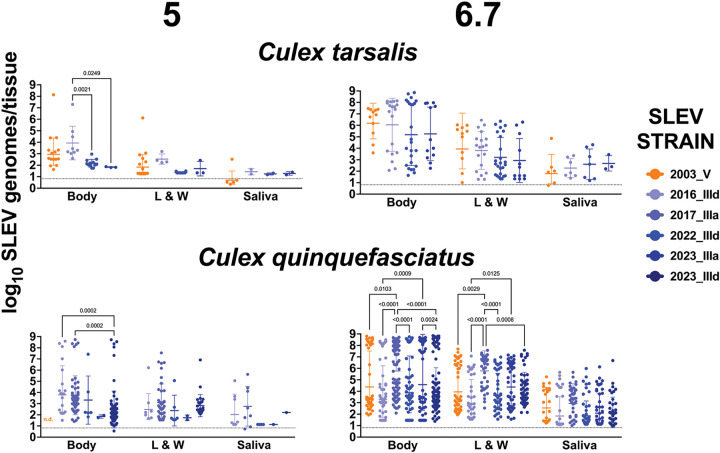
SLEV RNA levels 13,14, or 15 days post-bloodmeal in individual *Culex tarsalis* and *Culex quinquefasciatus* mosquitoes that ingested 5 or 6.7 log_10_ PFU/mL of different SLEV strains. L & W represents legs & wings. Rates were not statistically significantly different unless indicated with a p-value (Two-way ANOVA, Šídák’s multiple comparisons test; only values with p ≤ 0.05 were considered significant). Solid horizontal lines show geometric means and whiskers show geometric standard deviations. The dotted line shows the mean limit of detection (LOD), 0.82 log_10_ genomes/tissue. The LOD for some samples with a detectable measurement was below the mean LOD, which explains why some samples are under the LOD line.

We quantified SLEV RNA levels in individual infected mosquitoes from both species (Figure 6). Mean titers decreased in the order bodies > legs & wings > saliva. In *Cx. tarsalis* at 5 log_10_ PFU/mL, mean RNA levels in bodies were significantly higher for 2016_IIId compared with 2022_IIId and 2023_IIIa (p = 0.0021 and p = 0.0249, respectively; two-way ANOVA with Šídák’s multiple-comparisons test). No significant differences in mean RNA levels were detected among strains in legs and wings or saliva at this dose. At 6.7 log_10_ PFU/mL, mean RNA levels in *Cx. tarsalis* did not differ significantly among strains in any tissue type.

In *Cx. quinquefasciatus* at 5 log_10_ PFU/mL, mean RNA levels in bodies were significantly higher for 2016_IIId and 2017_IIIa compared with 2023_IIId (p = 0.0002 for both, two-way ANOVA with Šídák’s multiple-comparisons test) SLEV strain. At 6.7 log_10_ PFU/mL, significant differences were observed across multiple strains in bodies and legs and wings. Mean RNA levels for 2017_IIIa were significantly higher than all other strains (p ≤ 0.05, two-way ANOVA with Šídák’s multiple-comparisons test). Strain 2023_IIIa produced higher mean RNA levels than 2016_IIId and 2023_IIId (p ≤ 0.05, two-way ANOVA with Šídák’s multiple-comparisons test). No differences in mean RNA levels were detected in saliva from any strain at either dose.

## DISCUSSION

SLEV reemergence in California after 2015 represents a striking departure from historical patterns, in which SLEV activity was sporadic, spatially limited, and often disappeared for years following introduction. The persistence and geographic expansion of genotype III SLEV across many California counties from 2015–2025 raises questions about whether adaptation to local *Cx. tarsalis* and *Cx. quinquefasciatus* vectors may be contributing to this altered geographic pattern. By comparing contemporary California SLEV strains with a historical genotype V strain from 2003 that circulated prior to WNV invasion and the 11 year disappearance of SLEV statewide from 2004–2014, our study provides the first experimental evidence evaluating vector competence of post-2015 SLEV.

The five genotype III strains we evaluated showed equal or greater infectivity than the historical 2003 genotype V strain in *Cx. quinquefasciatus*, particularly at the mid-range bloodmeal dose of 5 log_10_ PFU/mL. Notably, *Cx. quinquefasciatus* exposed to the 2003 strain at this dose did not become infected; by contrast, each genotype III strain produced measurable infection and, for some strains, dissemination and transmission. This differential suggests that contemporary SLEV lineages may be better suited to infect *Cx. quinquefasciatus* under ecologically relevant viremia levels typical of passerine avian reservoirs, which range from 2–6 log_10_ PFU/ml (35). Given that *Cx. quinquefasciatus* is abundant in urban areas of southern and central California (36, 37) which continue to report much of the SLEV activity in mosquitoes in California (8), enhanced vector competence in this species may facilitate sustained and geographically widespread transmission.

In contrast, *Cx. tarsalis* displayed more heterogeneous and strain-specific responses to SLEV. Some genotype III strains (notably 2016_IIId and 2022_IIId) showed higher infection rates than the 2003 strain at high bloodmeal doses, but these differences did not extend consistently to dissemination or transmission, and no clear hierarchy of fitness emerged among strains or related to genetic classification within genotype III or detection year. This complexity mirrors earlier work on WNV in California, where viral fitness differences in *Cx. tarsalis* were sometimes inconsistent across strains and relatively small compared to those observed in avian hosts (29, 30). Taken together, our results suggest that while *Cx. tarsalis* remains a competent vector for both historical and contemporary SLEV, the enhanced fitness of genotype III strains is more evident, and potentially more epidemiologically consequential, in *Cx. quinquefasciatus*.

Another important outcome is the absence of a temporal fitness trend among post-2015 SLEV strains. Despite concerns that ongoing circulation of SLEV since 2015 might select for increasingly infectious genotypes, our data do not support a directional shift in viral fitness in California vector mosquitoes. Instead, fitness differences among genotype III strains appear idiosyncratic and not associated with year of detection. For example, the two IIIa strains (2017_IIIa and 2023_IIIa) differed markedly in their ability to infect *Cx. quinquefasciatus* at 5 log_10_ PFU/mL; the three IIId strains also displayed variable infection profiles. This pattern indicates that genetic variation within genotype III SLEV may influence vector competence at a fine scale, but no single lineage among the strains we evaluated exhibits uniformly elevated transmission potential. Furthermore, the lack of shared mutations in SLEV strains with higher vector fitness suggests that varying vector competence is likely driven by factors other than viral genetic differences in the SLEV strains evaluated.

This study has several limitations. We used long-established laboratory colonies of *Cx. tarsalis* and *Cx. quinquefasciatus* that may not fully capture the genetic or ecological variation present in wild mosquitoes in California. Differences in local adaptation or hybridization (mating between individuals from two genetically distinct populations) within the *Cx. pipiens* complex could influence infection or transmission outcomes for *Cx. quinquefasciatus* (which is part of the complex) in the field. Although the SLEV strains evaluated represent the predominant genotype III lineages detected since 2015, little sequencing of SLEV strains in California has been performed since 2018, which limits our ability to assess whether the genetic similarity of sublineages from 2022 and 2023 (IIIa, IIId, e.g.) we evaluated represents the genetic diversity of circulating viruses; additional recent strains may display distinct vector fitness patterns. The vector competence experiments were performed under controlled temperature and humidity conditions that do not reflect the environmental fluctuations experienced by mosquitoes in natural settings; temperature (38) and temperature variation (39) can alter infection dynamics. We quantified SLEV RNA rather than infectious virus in mosquito tissues and saliva which may overestimate transmission potential if RNA detection does not correspond to infectious virus (40). Finally, although dose-response experiments improve ecological relevance, experimentally delivered bloodmeal titers may not capture the full range of viremias in avian hosts during natural SLEV transmission cycles; our (41) and others’ (42) work show that artificial bloodmeals may overestimate or mis-represent true infectivity of vertebrate reservoirs for mosquito infection by arboviruses.

Our findings have implications for understanding SLEV ecology in California. The enhanced ability of multiple genotype III strains to infect *Cx. quinquefasciatus* may help explain why SLEV circulation has been concentrated in regions where this species is locally abundant, including the Coachella and Central Valleys. Even so, bird community composition, WNV-SLEV interactions (including cross-protection in avians), and climate-driven changes in mosquito abundance, likely play major roles in shaping SLEV transmission dynamics given that SLEV amplification is sensitive to host immunity, drought conditions, and patterns of mosquito feeding behavior.

This study provides phenotypic context for the genetic diversification of genotype III SLEV in the western United States. As multiple genotype III lineages continue to circulate, defining how genetic variation translates into differences in mosquito fitness is important for forecasting areas of SLEV transmission and human disease risk. Future work based on more SLEV genomic data will integrate analyses of specific viral mutations that affect vector fitness and avian competence. Mixed-virus strain competition infections in mosquito vectors and avian reservoirs to minimize inter-host variability is also planned to further elucidate virological determinants underlying SLEV persistence in California in the post-WNV era.

## Figures and Tables

**Table 1.** SLEV strains used.

Name	Strain identifier	Location	Year	Host Source	Passage history	GenBank Accession number	Genotype
2003_V	IMP115	Imperial County, CA	2003	*Cx. tarsalis*	Vero P3	PX678850	V
2016_IIId	KERN345	Kern County, CA	2016	*Cx. quinquefasciatus*	Vero P4	MN233315	III d
2017_IIIa	IMPR570	Imperial County, CA	2017	*Cx. tarsalis*	Vero P3	MN233313	III a
2022_IIId	FRWS339	Fresno West Side, CA	2022	*Cx tarsalis*	Vero P2	PX678851	III d
2023_IIIa	COAV3764	Coachella Valley, CA	2023	*Cx tarsalis*	Vero P2	PX678852	III a
2023_IIId	MERC702	Merced, CA	2023	*Cx tarsalis*	Vero P2	PX678853	III d

**Table 2:** Genome wide nucleotide / amino acid identity between SLEV strains used.

	2003_V	2016_IIId	2017_IIIa	2022_IIId	2023_IIIa	2023_IIId
**2003_V**	100.0 / 100.0	93.04 / 98.69	93.03 / 98.75	93.15 / 98.72	93.07 / 98.72	93.16 / 98.69
**2016_IIId**	93.04 / 98.69	100.0 / 100.0	99.66 / 99.77	99.61 / 99.74	99.50 / 99.71	99.38 / 99.65
**2017_IIIa**	93.03 / 98.75	99.66 / 99.77	100.0 / 100.0	99.45 / 99.74	99.35 / 99.71	99.57 / 99.77
**2022_IIId**	93.15 / 98.72	99.61 / 99.74	99.45 / 99.74	100.0 / 100.0	99.79 / 99.80	99.20 / 99.68
**2023_IIIa**	93.07 / 98.72	99.50 / 99.71	99.35 / 99.71	99.79 / 99.80	100.0 / 100.0	99.10 / 99.65
**2023_IIId**	93.16 / 98.69	99.38 / 99.65	99.57 / 99.77	99.20 / 99.68	99.10 / 99.65	100.0 / 100.0

**Table 3:** Primers used for SLEV RT-qPCR.

Primer Name	Sequence 5’ to 3’
SLE2420 (forward)	CTGGCTGTCGGAGGGATTCT
SLE2487c (reverse)	TAGGTCAATTGFACATCCCG
SLE2444-probe (probe)	FAM-TCTGGCGACCAGCGTGCAAGCCG-BHQ

FAM is fluorescein amidite and BHQ is black hole quencher.

**Table 4.** Statistical comparisons of rates of infection (bodies), dissemination (egs & wings) and transmission (saliva) in *Culex tarsalis* exposed to SLEV.

5 log_10_ PFU/mL SLEV bloodmeal									
	INFECTION			DISSEMINATION			TRANSMISSION		
SLEV strain comparison	Odds Ratio	Raw p	FDR adj p	Odds Ratio	Raw p	FDR adj p	Odds Ratio	Raw p	FDR adj p
2003_V vs 2016_IIId	0.96	0.0617	0.1029	1.17	1	1	3.13	0.5758	1
2003_V vs 2022_IIId	1.55	0.368	0.441	4.38	0.217	0.993	2.5	1	1
2003_V vs 2023_IIIa	9.14	0.0004	**0.0011**	1.75	1	1	0	1	1
2016_IIId vs 2022_IIId	1.62	0.3389	0.3677	3.75	0.331	0.993	0.8	1	1
2016_IIId vs 2023_IIIa	9.51	0.0686	0.1029	1.5	1	1	0	1	1
2022_IIId vs 2023_IIIa	5.89	0.0054	**0.0107**	0.4	0.516	1	0	1	1
**6.7 log_10_ PFU/mL SLEV bloodmeal**									
2003_V vs 2016_IIId	0.24	0.0103	**0.0207**	1.88	1	1	1.93	0.688	0.83
2003_V vs 2022_IIId	0.28	0.0174	**0.0261**	0.87	1	1	3.43	0.139	0.83
2003_V vs 2023_IIIa	1.33	0.62	0.744	3.33	0.59	1	3	0.37	0.83
2016_IIId vs 2022_IIId	1.18	0.799	0.799	0.46	0.638	1	1.78	0.503	0.83
2016_IIId vs 2023_IIIa	5.63	0.0013	**0.0039**	1.78	0.653	1	1.56	0.691	0.83
2022_IIId vs 2023_IIIa	4.76	0.001	**0.0039**	3.83	0.304	1	0.88	1	1

Fisher’s exact tests employing FDR Benjamini–Hochberg corrections. Bold indicates p values ≤ 0.05.

**Table 5.** Statistical comparisons of rates of infection (bodies), dissemination (legs & wings) and transmission (saliva) in *Culex quinquefasciatus* exposed to SLEV.

5 log_10_ PFU/mL SLEV bloodmeal									
	INFECTION			DISSEMINATION			TRANSMISSION		
Comparison	Odds Ratio	Raw p	FDR adj p	Odds Ratio	Raw p	FDR adj p	Odds Ratio	Raw p	FDR adj p
2003_V vs 2016_IIId	0	4.05E-08	**6.76E-08**	n.d.	n.d.	n.d.	n.d.	n.d.	n.d.
2003_V vs 2017_IIIa	0	1.34E-27	**1.01E-26**	n.d.	n.d.	n.d.	n.d.	n.d.	n.d.
2003_V vs 2022_IIId	0	0.001	**0.001475**	n.d.	n.d.	n.d.	n.d.	n.d.	n.d.
2003_V vs 2023_IIIa	0	0.20	0.214736	n.d.	n.d.	n.d.	n.d.	n.d.	n.d.
2003_V vs 2023_IIId	0	4.77E-31	**7.16E-30**	n.d.	n.d.	n.d.	n.d.	n.d.	n.d.
2016_IIId vs 2017_IIIa	0.1	6.63E-09	**1.24E-08**	0.101	0.000	**0.004**	1	1.00	1.00
2016_IIId vs 2022_IIId	2.7	0.064144	**0.080181**	0.291	0.378	0.699	6.7	0.27	0.57
2016_IIId vs 2023_IIIa	13.7	4.73E-05	**7.09E-05**	0.727	1.000	1.000	∞	0.444	0.63
2016_IIId vs 2023_IIId	0.1	2.05E-09	**4.4E-09**	0.571	0.419	0.699	45	0.001	**0.004**
2017_IIIa vs 2022_IIId	31.4	5.42E-13	**1.36E-12**	2.880	0.267	0.668	6.7	0.27	0.57
2017_IIIa vs 2023_IIIa	158.9	1.82E-21	**6.84E-21**	7.200	0.262	0.668	∞	0.44	0.63
2017_IIIa vs 2023_IIId	1.3	0.67	0.67039	5.657	0.001	**0.006**	45	0.001	**0.004**
2022_IIId vs 2023_IIIa	5.1	0.07	**0.084423**	2.500	1.000	1.000	∞	1.00	1.00
2022_IIId vs 2023_IIId	0.0	3.23E-14	**9.69E-14**	1.964	0.687	0.981	6.75	0.28	0.57
2023_IIIa vs 2023_IIId	0.0	8.9E-24	**4.45E-23**	0.786	1.000	1.000	0	1.00	1.00
**6.7 log_10_ PFU/mL SLEV bloodmeal**									
2003_V vs 2016_IIId	1.26	0.66	0.71	∞	**0.00968**	**0.048**	1.25	0.661	0.826
2003_V vs 2017_IIIa	0	8.49×10^−7^	**3.18×10^−6^**	—	1	1	0.28	**0.0055**	**0.0194**
2003_V vs 2022_IIId	0.58	0.33	0.48	∞	1	1	0.55	0.211	0.316
2003_V vs 2023_IIIa	0.78	0.64	0.71	∞	0.471	0.742	0.25	**0.0065**	**0.0194**
2003_V vs 2023_IIId	0	1.05×10^−7^	**5.26×x10^−7^**	—	1	1	0.32	**0.0109**	**0.0273**
2016_IIId vs 2017_IIIa	0	5.43×10^−8^	**4.07×10^−7^**	0	**0.00736**	**0.048**	0.23	**0.0028**	**0.0179**
2016_IIId vs 2022_IIId	0.46	0.16	0.26	0.13	0.0457	0.171	0.44	0.0787	0.169
2016_IIId vs 2023_IIIa	0.61	0.35	0.48	0.15	0.109	0.328	0.2	**0.0019**	**0.0179**
2016_IIId vs 2023_IIId	0	4.67×10^−9^	**7.00×10^−8^**	0	**0.00736**	**0.048**	0.25	**0.0036**	**0.0179**
2017_IIIa vs 2022_IIId	∞	1.95×10^−4^	**3.66×10^−4^**	∞	0.495	0.742	1.94	0.181	0.302
2017_IIIa vs 2023_IIIa	∞	2.67×10^−5^	**6.68×10^−5^**	∞	0.451	0.742	0.89	1	1
2017_IIIa vs 2023_IIId	—	1.00	1.00	—	1	1	1.12	1	1
2022_IIId vs 2023_IIIa	1.34	0.60	0.71	1.2	1	1	0.46	0.155	0.29
2022_IIId vs 2023_IIId	0	5.42×10^−5^	**1.16×10^−4^**	0	0.495	0.742	0.58	0.27	0.368
2023_IIIa vs 2023_IIId	0	5.91×10^−6^	**1.77×10^−5^**	0	0.451	0.742	1.26	0.8	0.923

Fisher’s exact tests employing FDR Benjamini–Hochberg corrections. Bold indicates p values ≤ 0.05.

**Table 6:** Amino acid differences (N=60) across 6 SLEV strains used for this study.

Gene	Amino Acid Position	2003_V	2016_IIId	2017_IIIa	2022_IIId	2023_IIIa	2023_IIId
C	10	R	K	K	K	K	K
C	71	A	A	V	A	A	A
C	92	L	M	L	L	L	L
C	114	A	A	A	V	A	A
C	116	I	I	I	T	I	I
prM/M	261	A	V	V	V	V	V
E	340	D	E	E	E	E	E
E	352	T	S	S	S	S	S
E	450	N	N	N	N	S	N
NS1	796	D	N	D	D	D	D
NS1	859	N	S	S	S	S	S
NS1	862	R	K	K	K	K	K
NS1	885	H	Y	Y	Y	Y	Y
NS1	887	R	K	K	K	K	K
NS1	891	Q	R	R	R	R	R
NS1	901	D	N	N	N	N	N
NS1	963	G	E	E	E	E	E
NS1	1077	T	T	T	T	I	T
NS2A	1236	L	F	F	F	F	F
NS2A	1250	I	V	I	I	I	I
NS2A	1291	C	Y	Y	Y	Y	Y
NS2A	1295	I	I	I	I	F	I
NS2A	1347	A	A	A	A	V	A
NS2B	1416	K	R	R	R	R	R
NS3	1837	A	A	A	T	A	A
NS3	1853	S	N	N	N	N	N
NS3	1898	K	K	K	K	R	K
NS3	1978	N	N	S	N	N	N
NS3	2049	V	V	V	A	V	A
NS3	2088	R	K	K	K	K	K
NS3	2093	R	K	K	K	K	K
NS4A	2184	T	T	T	T	T	A
NS4A	2195	G	S	S	S	S	S
NS4A	2210	L	S	S	S	S	S
2K	2288	G	G	E	G	E	G
NS4B	2289	S	P	P	P	P	P
NS4B	2292	F	F	F	F	F	S
NS4B	2298	P	S	P	S	P	S
NS4B	2301	D	X*	X*	D	D	D
NS4B	2333	Q	Q	Q	Q	Q	H
NS4B	2344	A	S	S	S	S	S
NS4B	2396	V	I	I	I	I	I
NS4B	2419	A	V	A	A	A	A
NS4B	2514	V	I	I	I	I	I
NS5	2650	Y	H	H	H	H	H
NS5	2693	T	A	A	A	A	A
NS5	2702	T	I	I	I	I	I
NS5	2706	I	V	V	V	V	V
NS5	2713	M	T	T	T	T	T
NS5	2940	L	F	F	F	F	F
NS5	2967	E	K	K	K	K	K
NS5	2989	M	T	T	T	T	T
NS5	3026	N	S	S	S	S	S
NS5	3031	H	Y	Y	Y	Y	Y
NS5	3120	V	I	I	I	I	I
NS5	3162	V	I	I	I	I	I
NS5	3200	I	T	T	T	T	T
NS5	3279	K	R	R	R	R	R
NS5	3414	I	V	V	V	V	A
NS5	3430	V	A	A	A	A	A

Grey shading shows the minority amino acid.

* X denotes incomplete sequence in GenBank record.

C is capsid, prM is premembrane, M is membrane, E is envelope, NS is non-structural.
